# Supplementary material for: Fully chemical interface engineering for statically and dynamically stable perovskite solar cells
Source: Nat Commun. 2025 Sep 29;16:8575. doi: 10.1038/s41467-025-63588-8 (PMC12480934; doi:10.1038/s41467-025-63588-8)
Supplement: Supplementary file 1 — Supplementary Information [file 41467_2025_63588_MOESM1_ESM.pdf]

Supplementary Information

Fully chemical interface engineering for statically and dynamically stable perovskite solar cells

Author Information

Luyao Li[1, 2], Cheng Wang[1], Weicun Chu[1], Jaewang Park[3], Yiming Dai[1], Qiankai Ba[4], Kaifeng Wang[4], Jiaxing Gao[1], Zeliang Wei[1], Xiaoming Zhao[1], Xuchen Nie[1], Lixiong Yin[2,]*, Sang Il Seok[3,]*, Riming Nie[1,]*, Wanlin Guo[1]

Affiliations

[1] State Key Laboratory of Mechanics and Control of Mechanical Structures, Key Laboratory for Intelligent Nano Materials and Devices of the Ministry of Education, Institute for Frontier Science, Nanjing University of Aeronautics and Astronautics, Nanjing 210016, PR China

[2] School of Materials Science and Engineering, Shaanxi University of Science and Technology, Xi'an, 710021, PR China

[3] Department of Energy Engineering, School of Energy and Chemical Engineering, Ulsan National Institute of Science and Technology, 50 UNIST-gil, Eonyang-eup, Ulju-gun, Ulsan 44919, Republic of Korea

[4] Shanghai Shengjian Technology Co., Ltd.

*Corresponding authors: rmnie@nuaa.edu.cn; ylx@sust.edu.cn; seoksi@unist.ac.kr

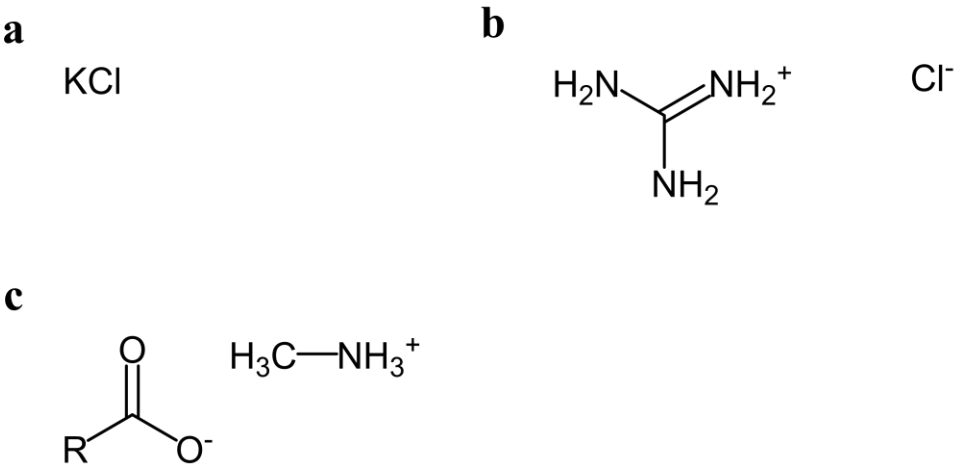

Supplementary Fig. 1. Chemical structure diagram of several typical modifiers. (a) KCl; (b) $CH_5N_3 \cdot HCl$; (c) MAAc.

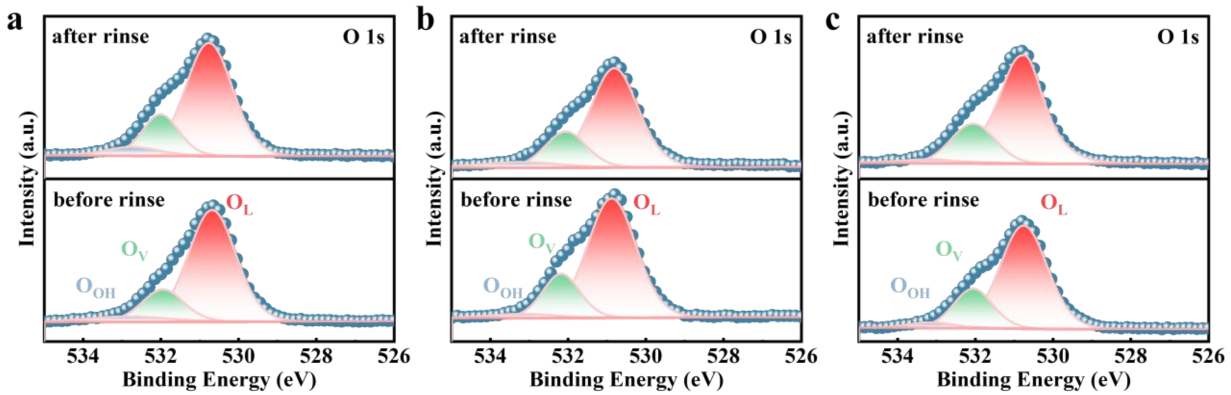

Supplementary Fig. 2. XPS spectra of each electrode before and after washing with DMF. O1s core level of (a) SnO_2/KCl, (b) SnO_2/Gua, (c) SnO_2/MAAc.

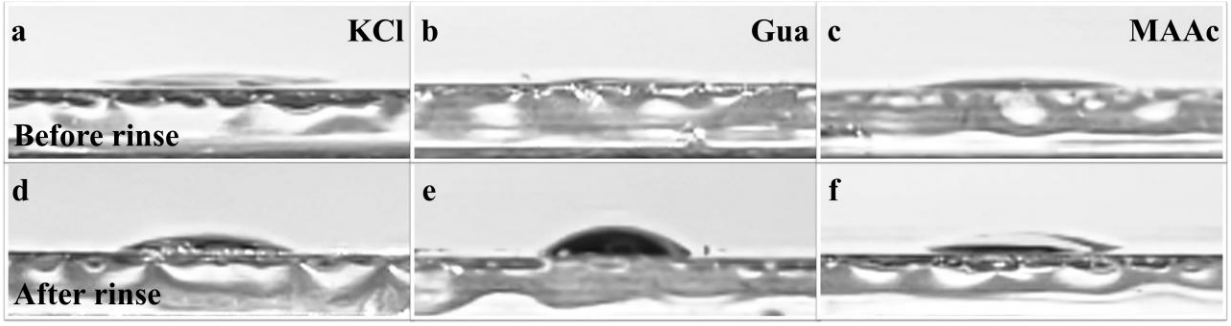

Supplementary Fig. 3. Contact angle test results before and after cleaning various oxide substrates with DMF.

The contact angle measurements were performed using ultrapure water droplets as the testing liquid. Supplementary Fig. 3 a-c present contact angle measurements for different SnO_2 substrates: (a) SnO_2/KCl, (b) SnO_2/Gua, and (c) SnO_2/MAAc. All substrates initially exhibited excellent wettability. These substrates were then subjected to extensive DMF washing using the following protocol: 1000 μL of DMF was deposited onto each SnO_2 substrate, allowed to stand for approximately 15 seconds for complete wetting, followed by spin-coating at 3000 rpm for 30 seconds, and finally heated at 150°C for 5 minutes to remove residual solvent.

Supplementary Fig. 3 d-f show the corresponding contact angles after intensive DMF washing for (d) SnO_2/KCl, (e) SnO_2/Gua, and (f) SnO_2/MAAc. Notably, all substrates demonstrated significantly increased contact angles post-washing. This phenomenon can be primarily attributed to: The hydrophilic/polar nature of the surface modifiers - their removal via DMF washing reduced the polar component of surface energy. Consequently, the decreased surface modifier content diminished the substrates' hydrophilicity, manifesting as increased contact angles.

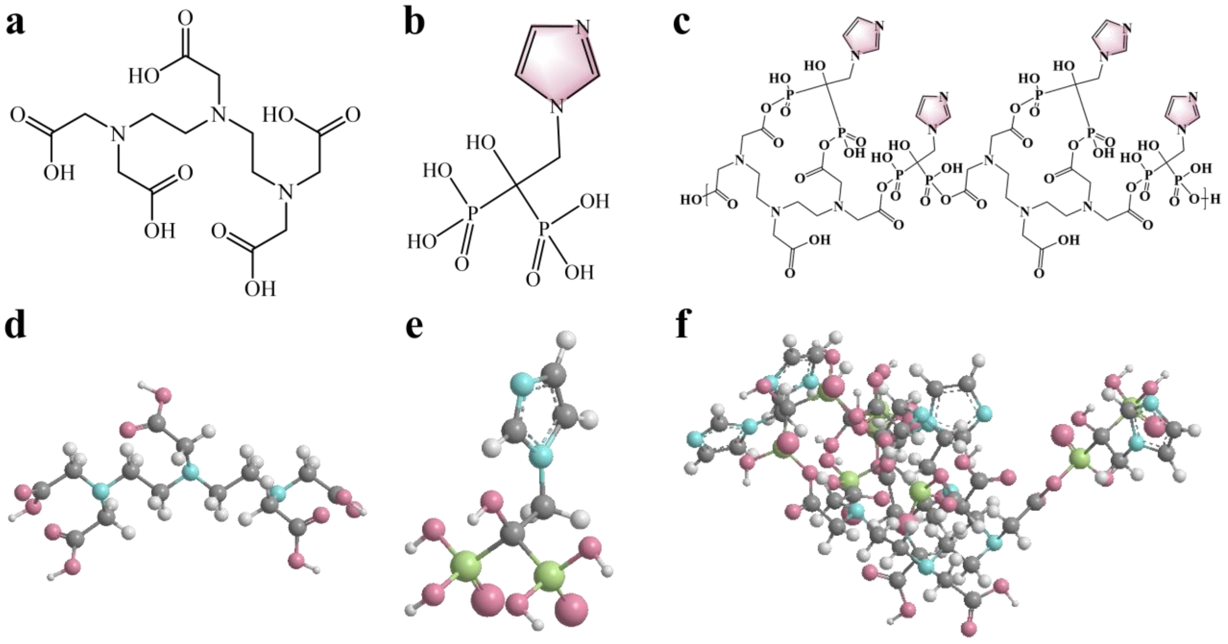

Supplementary Fig. 4. Chemical structure diagram of (a) DTPA; (b) Zol; (c) the reaction product of DTPA and Zol. (d-f) The corresponding molecular ball-and-stick model.

Conventional surface modifiers or interface optimization molecules typically interact with SnO_2 through only one or a few binding sites, which inherently limits their overall adsorption strength. In contrast, our DTPA and Zol modifiers undergo in situ crosslinking-like reactions that enable multiple DTPA molecules to form an extended network structure through Zol bridging. The interconnected molecular framework enables cooperative interactions among adjacent binding sites, thereby dramatically enhancing the overall adsorption capability of the modifier system.

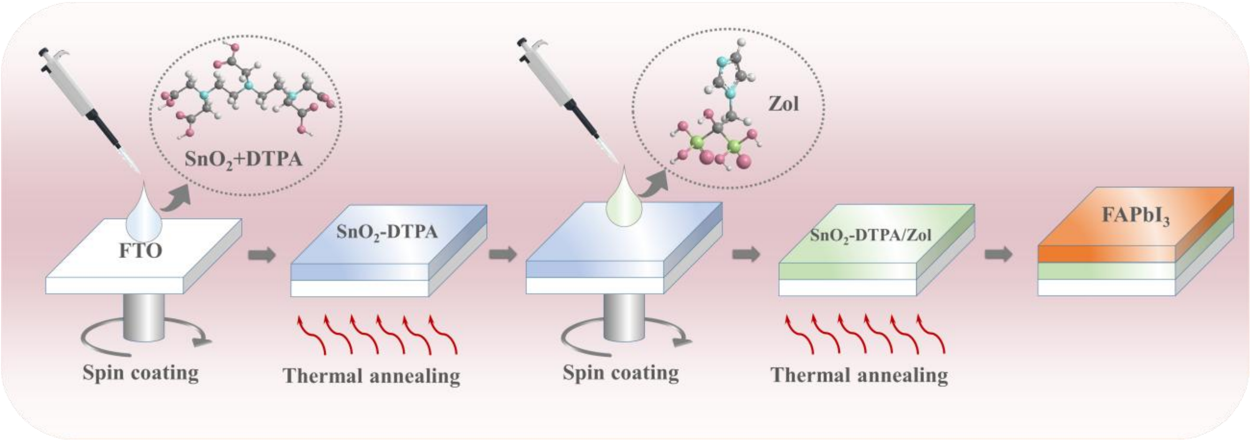

Supplementary Fig. 5. Schematic diagram of the preparation process for SnO_2 substrates modified with DTPA and Zol.

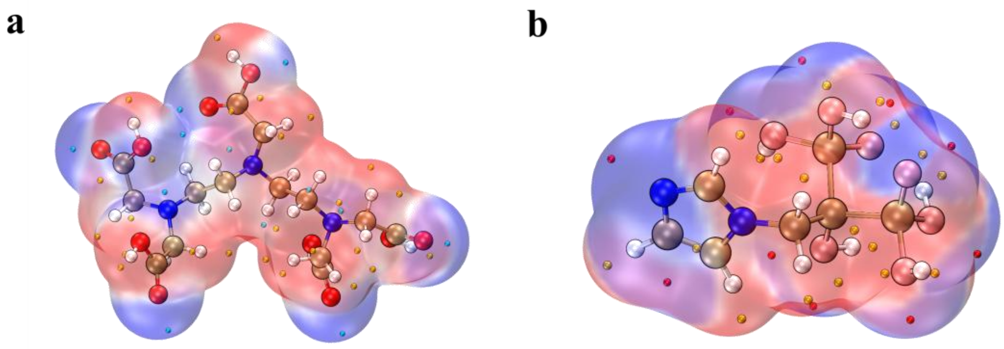

Supplementary Fig. 6. Electrostatic potential of (a) DTPA and (b) Zol.

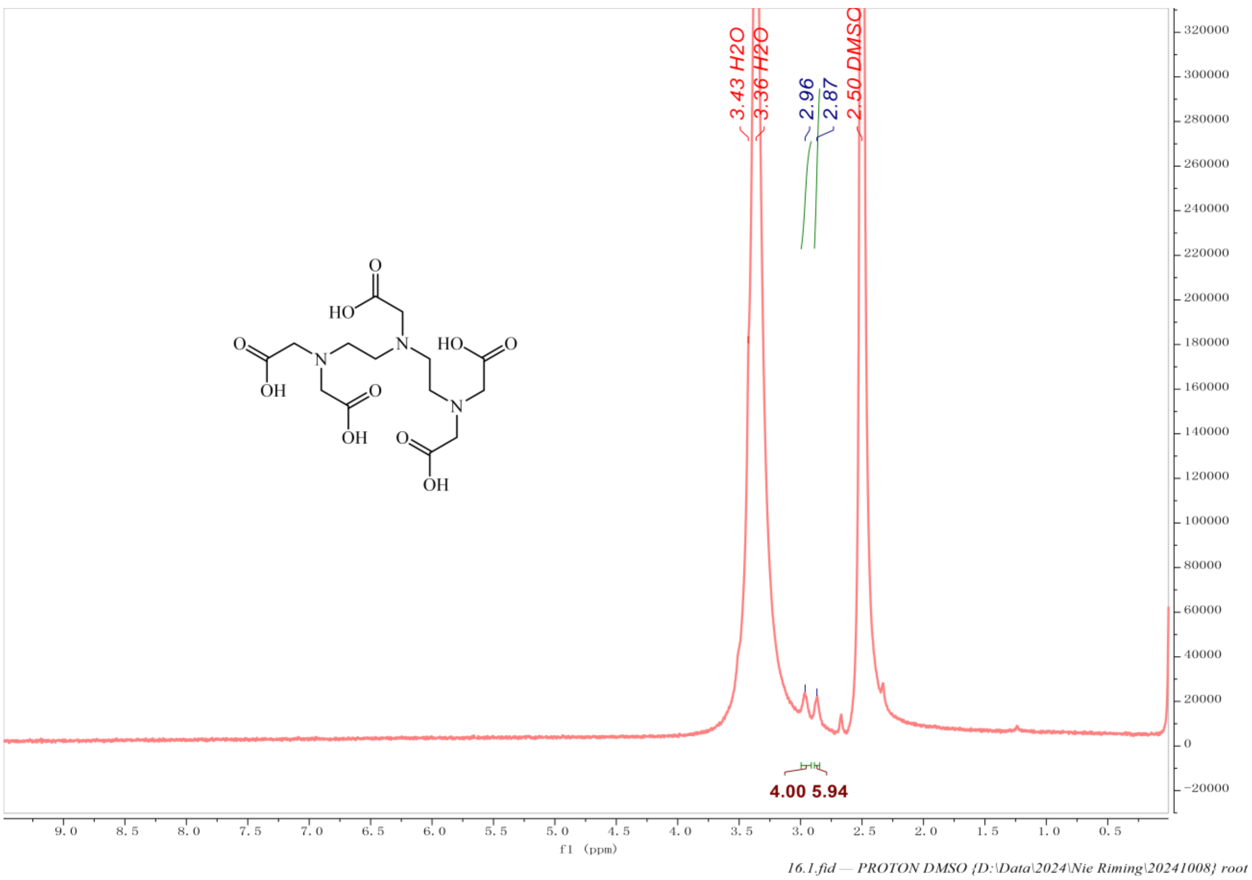

Supplementary Fig. 7. ^{1}H NMR spectrum of DTPA in DMSO-d_6.

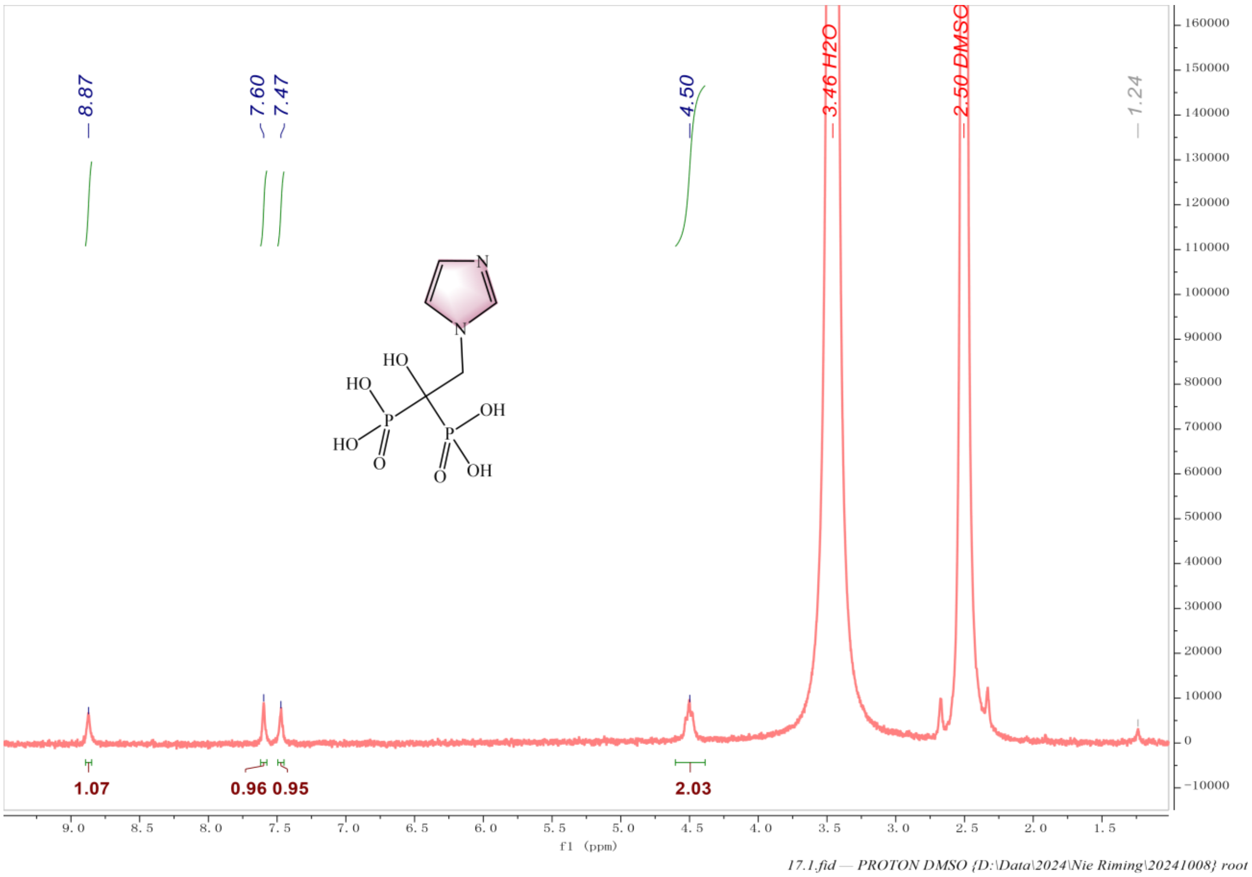

Supplementary Fig. 8. ^{1}H NMR spectrum of Zol in DMSO-d_6.

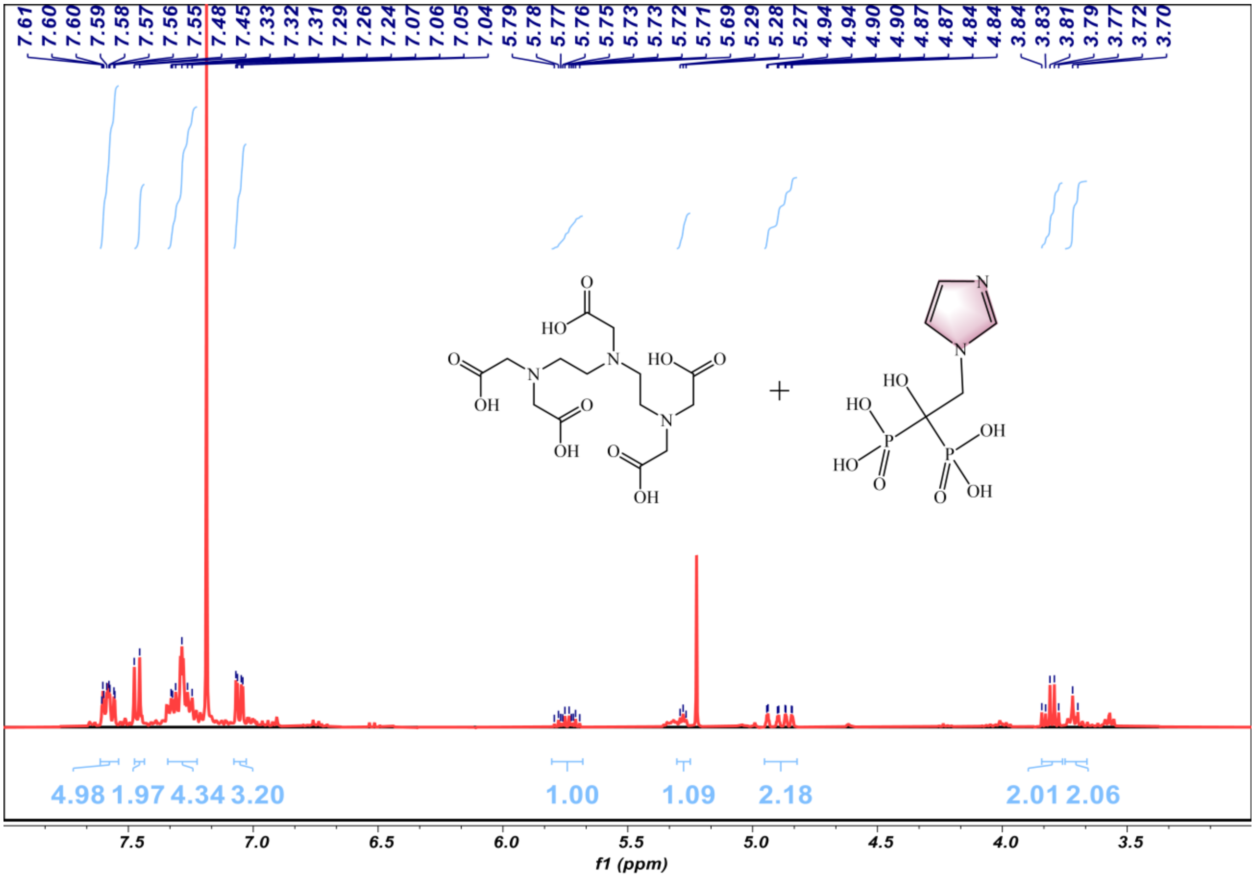

Supplementary Fig. 9. ^{1}H NMR spectrum of the reaction product of DTPA and Zol in DMSO-d_6.

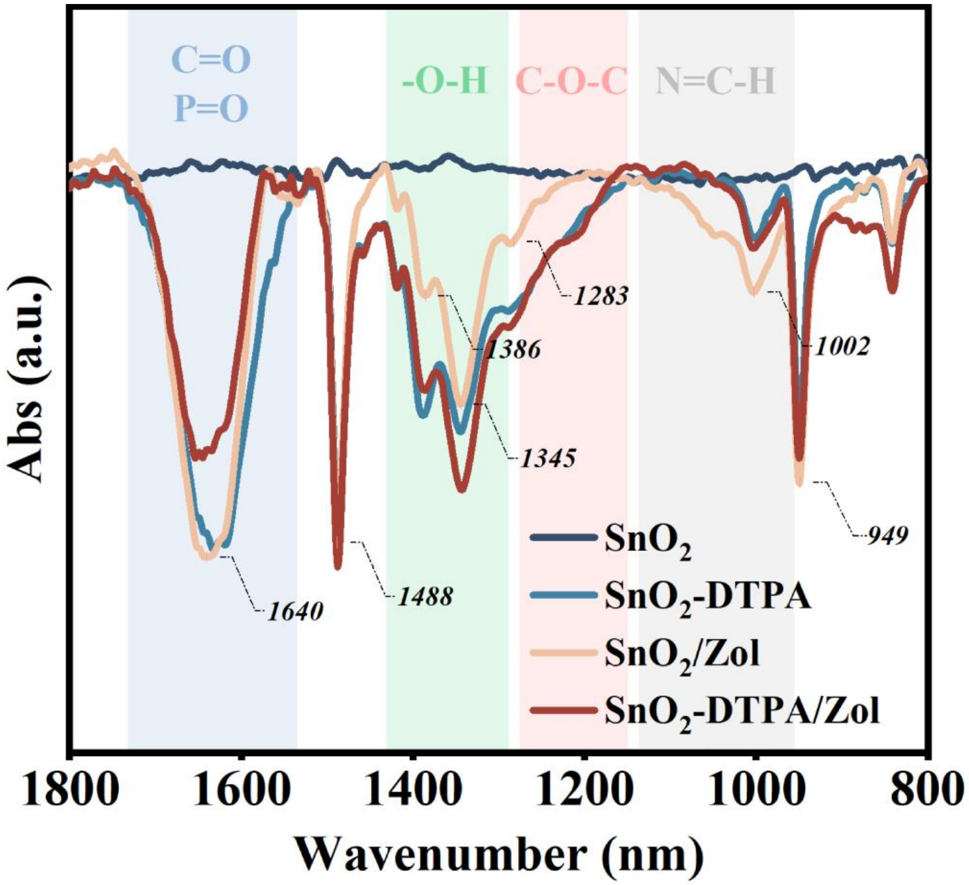

Supplementary Fig. 10. FTIR of pure SnO_2, SnO_2-DTPA, SnO_2/Zol and SnO_2-DTPA/Zol films.

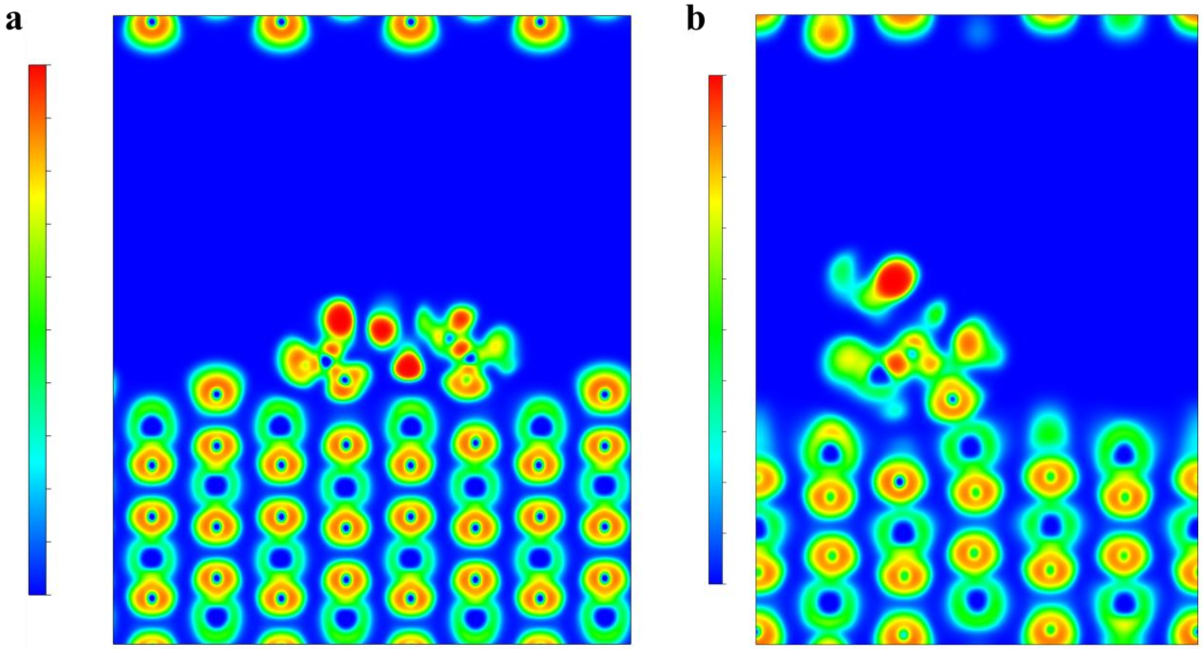

Supplementary Fig. 11. The ELF image of a) DTPA and b) Zol absorption on SnO_2 surface, obtained through DFT calculations.

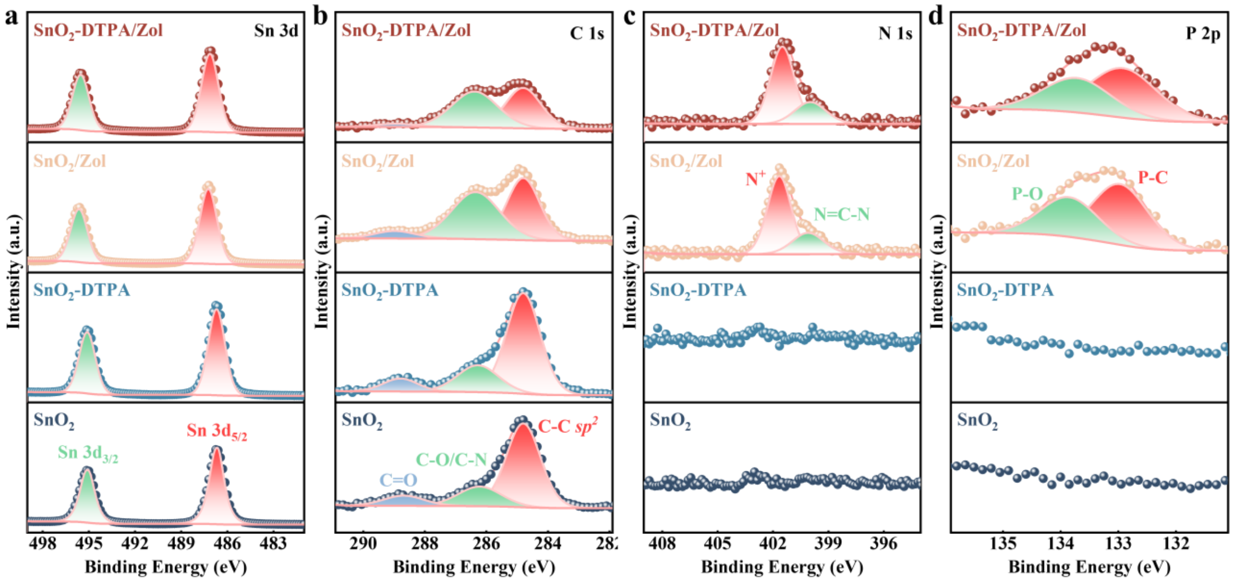

Supplementary Fig. 12. XPS spectra of pure SnO_2, SnO_2-DTPA, SnO_2/Zol and SnO_2-DTPA/Zol films for (a) Sn 3*d*; (b) C 1*s*; (c) N 1*s* and (d) P 2*p*, respectively.

Since DTPA molecules are uniformly dispersed throughout the entire SnO_2 layer with minimal additive amounts, and considering that XPS can only detect the chemical environment at the sample surface with limited instrument sensitivity, the N 1*s* peak from trace DTPA molecules on the SnO_2-DTPA sample surface remained undetectable. In contrast, Zol modifiers were exclusively concentrated at the SnO_2 surface with relatively higher local concentrations, enabling clear detection of distinct N 1*s* and P 2*p* peaks.

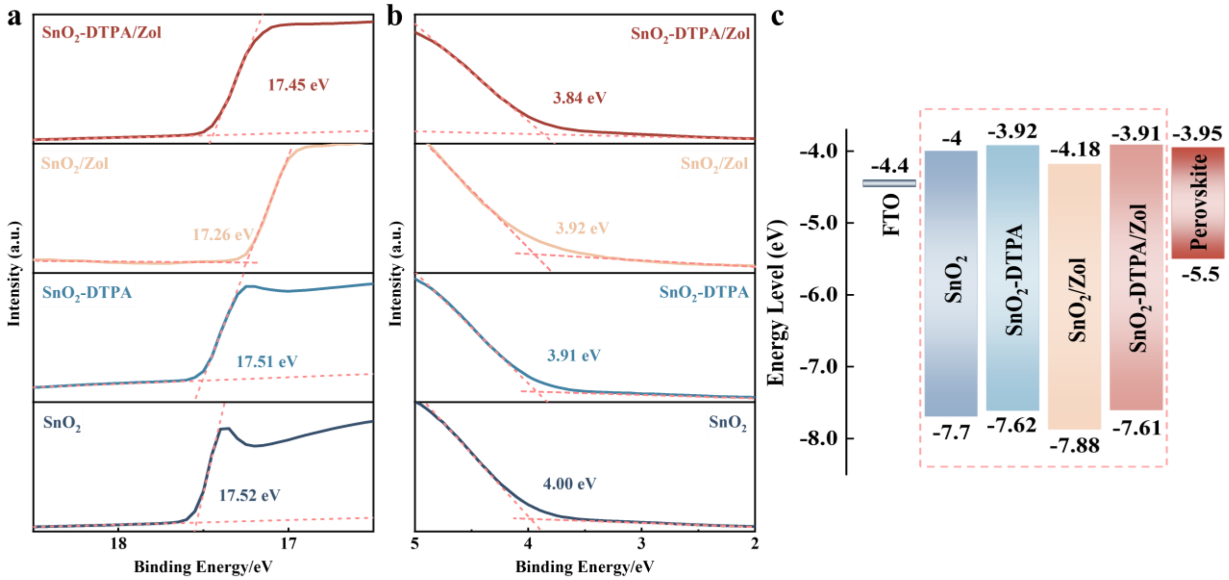

Supplementary Fig. 13. (a and b) UPS spectra of SnO_2, SnO_2-DTPA, SnO_2/Zol and SnO_2-DTPA/Zol films. (c) Schematic diagram of the energy level structure of various SnO_2 thin films.

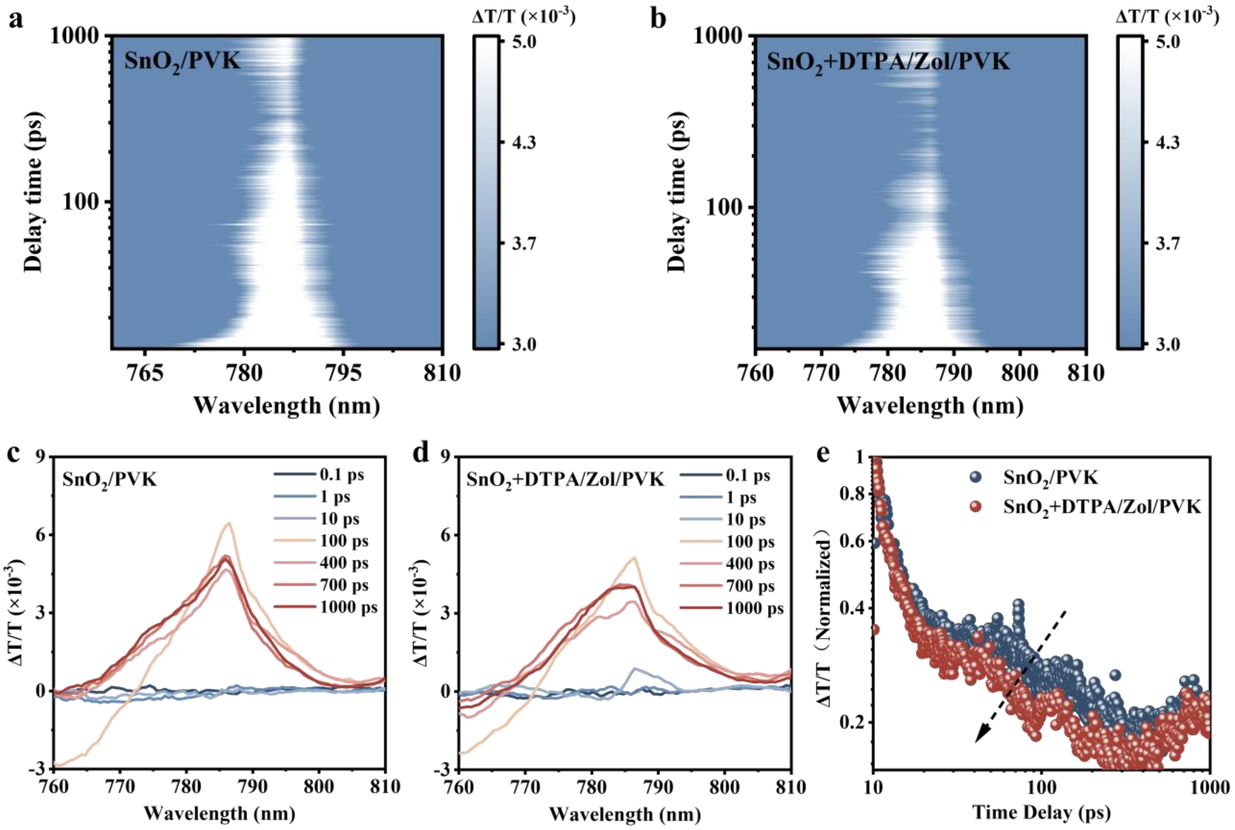

Supplementary Fig. 14. Femtosecond transient absorption spectroscopy (fs-TAS) of (a) SnO_2/PVK and (b) SnO_2-DTPA/Zol/PVK films. Corresponding TA spectra at different decay times of (c) SnO_2/PVK and (d) SnO_2-DTPA/Zol/PVK films. (e) Corresponding TA decay kinetics.

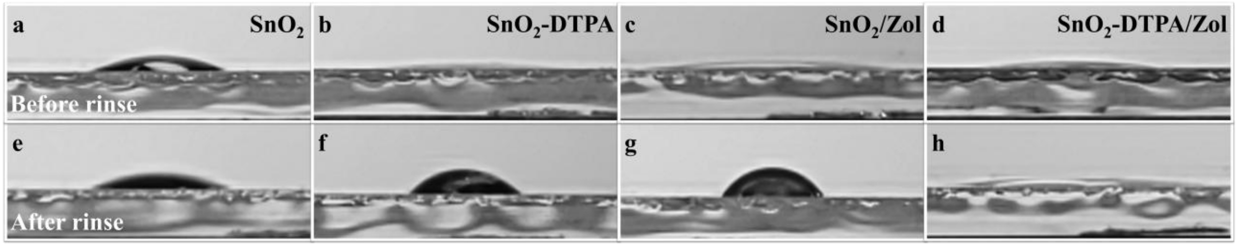

Supplementary Fig. 15. Contact angle test results before and after cleaning various SnO_2 films with DMF.

Contact angle measurements were performed using ultrapure water droplets as the test liquid. Fig. S15 a-d shows the contact angles of different SnO_2 substrates: (a) pure SnO_2, (b) SnO_2-DTPA, (c) SnO_2/Zol, and (d) SnO_2-DTPA/Zol. All modified SnO_2 substrates demonstrated better wettability than pure SnO_2. The substrates were washed with large amounts of DMF. Specifically, 1000 μL DMF was dropped onto different SnO_2 substrates, allowed to stand for about 15 seconds for complete wetting, then spin-coated at 3000 rpm for 30 seconds, and heated at 150℃ for 5 minutes to remove the solvent.

Fig. S15 e-h shows the corresponding contact angles of SnO_2 substrates after extensive DMF washing: (e) pure SnO_2, (f) SnO_2-DTPA, (g) SnO_2/Zol, and (h) SnO_2-DTPA/Zol. After extensive DMF washing: The contact angle of pure SnO_2 substrate showed almost no change, proving that DMF washing treatment has little effect on the wettability of SnO_2 itself. The contact angles of SnO_2-DTPA and SnO_2/Zol substrates increased significantly, indicating partial desorption of DTPA and Zol molecules from the SnO_2 surface. The contact angle of the SnO_2-DTPA/Zol sample showed almost no change and still exhibited good wettability. This proves that co-modification with DTPA and Zol can ensure that SnO_2 does not fail due to desorption under the load of upper film solvents.

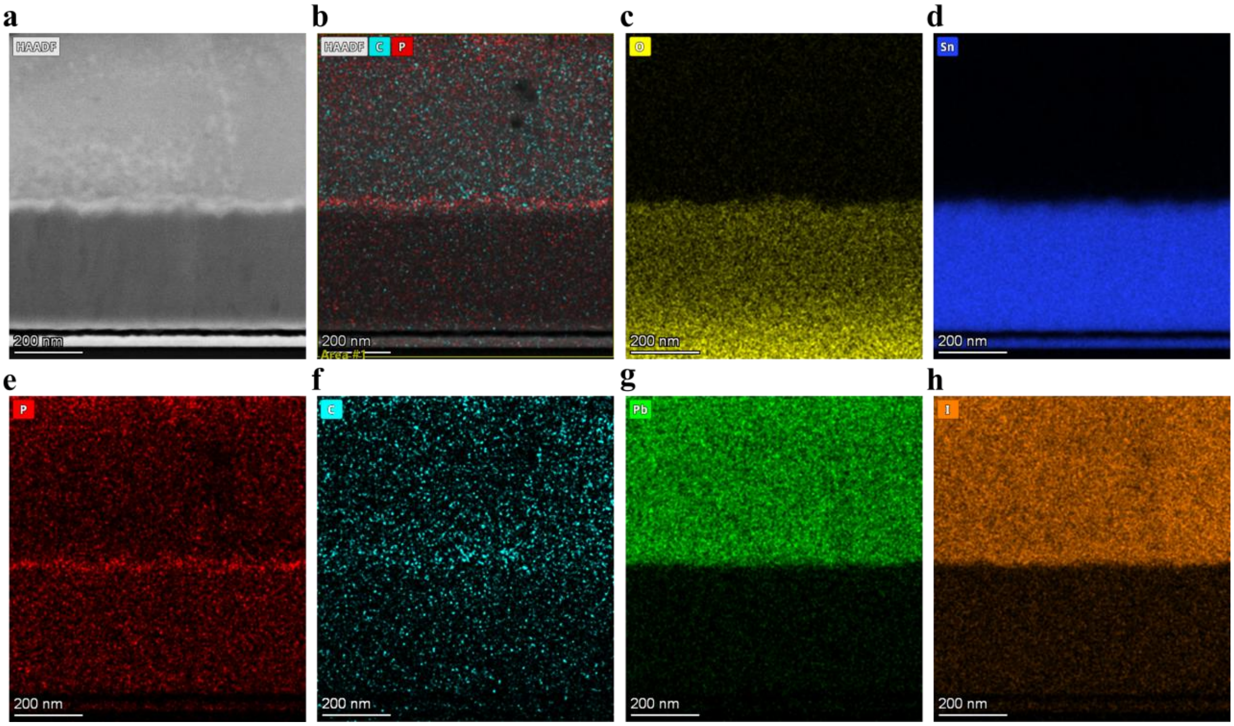

Supplementary Fig. 16. Cross-sectional HAADF and corresponding TEM-EDS image of glass/FTO/SnO_2-DTPA/Zol/PVK.

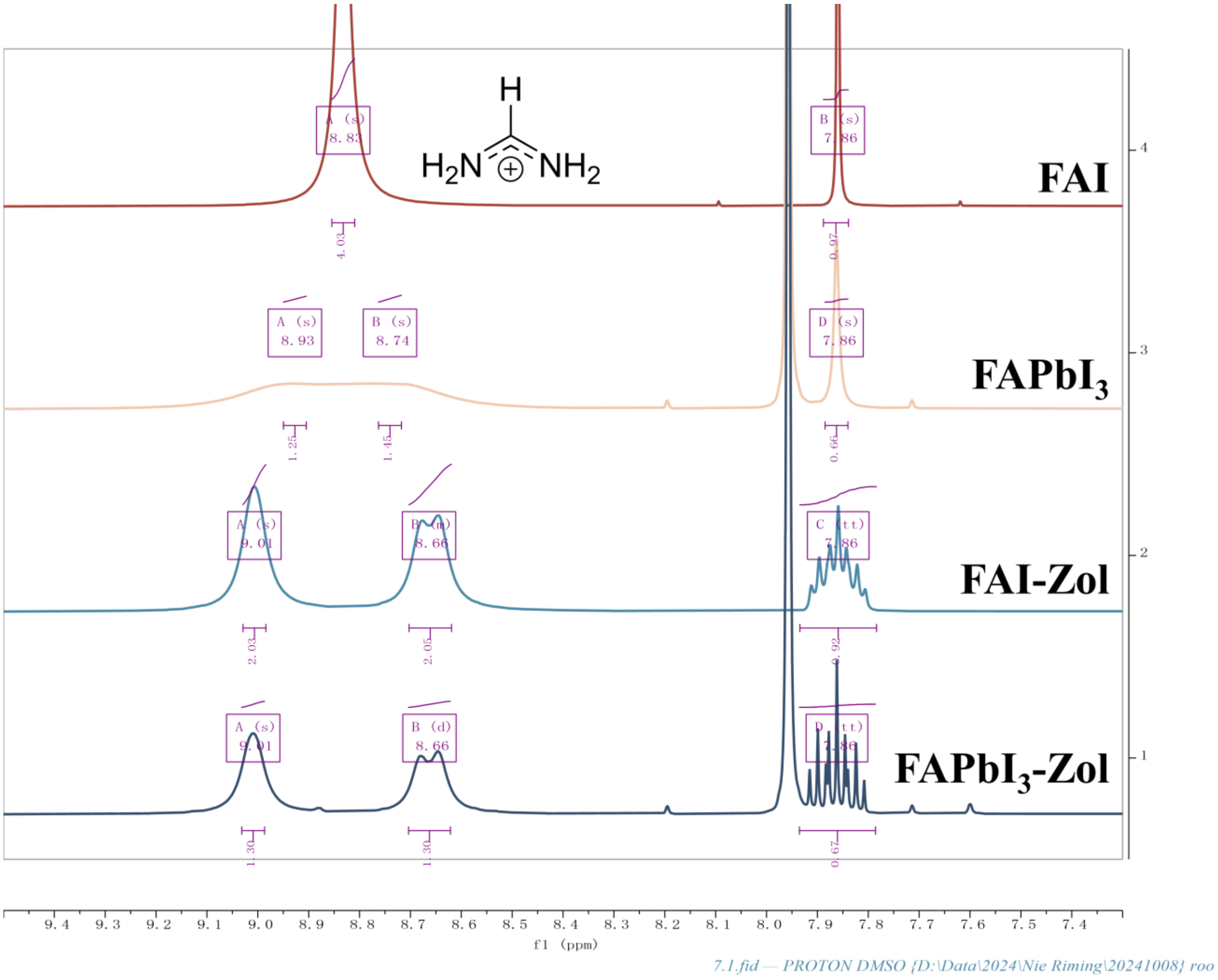

Supplementary Fig. 17. ^{1}H NMR spectrum of the FAI, $FAPbI_3$, FAI-Zol and $FAPbI_3$-Zol in DMSO-d_6.

1H NMR spectroscopy was employed to investigate the interaction between Zol and FAI (Supplementary Fig. 13). Upon modification of Zol, the hydrogen peak of FAI's -NH_2 group at 8.83 ppm split into two new peaks at 9.01 ppm and 8.66 ppm. Additionally, the two -NH_2 hydrogen peaks of $FAPbI_3$, originally at 8.93 ppm and 8.74 ppm respectively, shifted to 9.01 ppm and 8.66 ppm following Zol modification. As consistently demonstrated in Figures 3D and E, these observations confirm the formation of robust N-H ··N hydrogen bonding interactions between Zol and FAI.

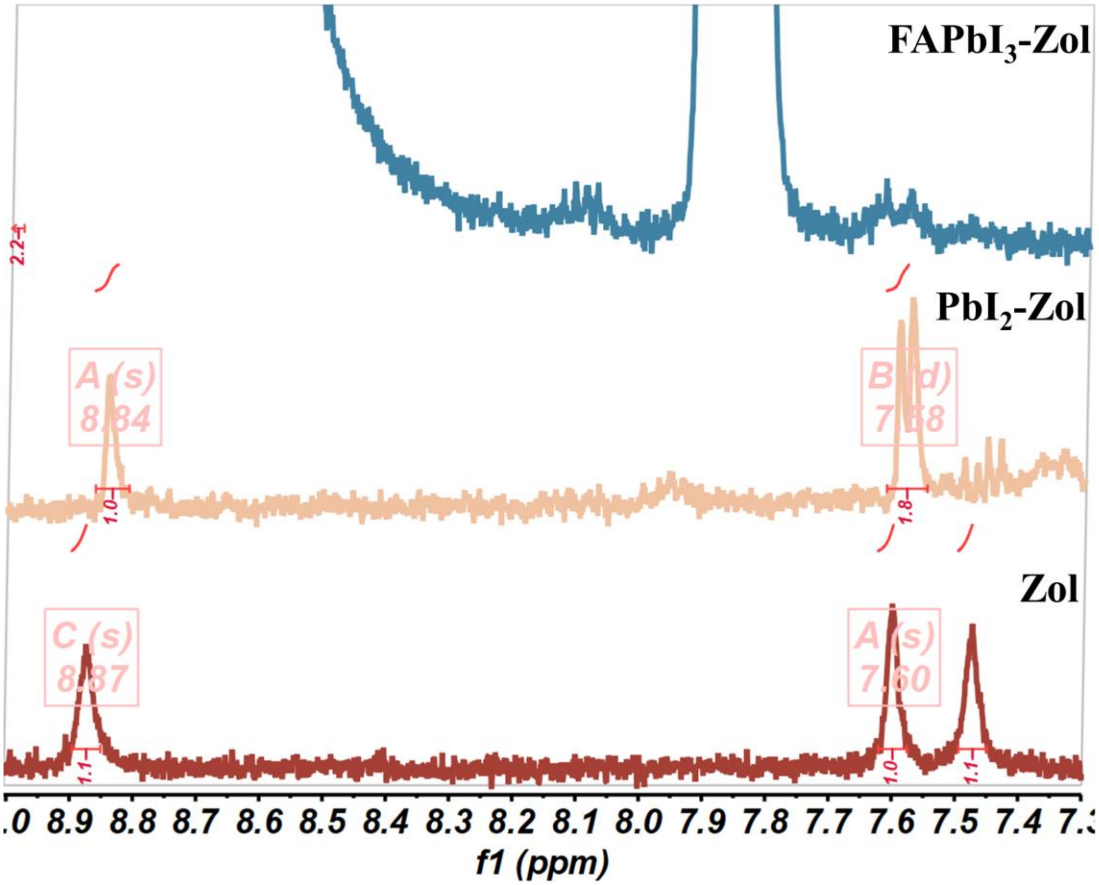

Supplementary Fig. 18. ^{1}H NMR spectrum of the Zol, PbI_2-Zol and $FAPbI_3$-Zol in DMSO-d_6.

1H-NMR was used to study the interaction between Zol and PbI_2 (Supplementary Fig. 14). Compared with pure Zol, shift of δ values were observed when Zol was mixed with PbI_2. The hydrogen peak of Zol at 8.87 ppm shifted to 8.84 ppm, the peak at 7.60 ppm split into two peaks, and the peak at 7.47 ppm disappeared. This is attributed to the interaction between Zol and PbI_2 distorting the electron density cloud around the hydrogen nuclei, which affects the magnetic field changes and consequently alters the shielding effect on the hydrogen atoms.

Therefore, we propose a dual mechanism: During PbI_2 deposition, Zol binds with Pb to promote ordered arrangement in the bottom PbI_2 layer. Meanwhile, during FAI deposition, Zol molecules on the substrate surface rapidly coordinate with FA^+. This leads to more ordered terminal atomic arrangement at the perovskite bottom, reduced defects, and enhanced crystallinity, which is consistent with the XRD results.

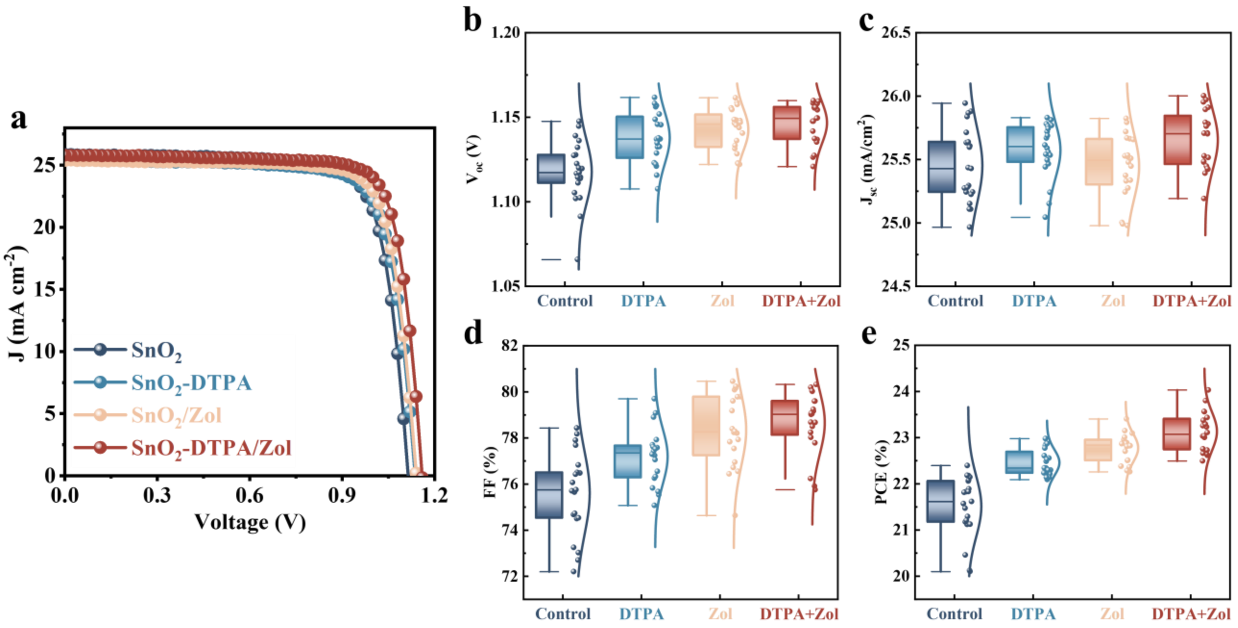

Supplementary Fig. 19. (a) *J*-*V* curves, (b) V_{oc}, (c) J_{sc}, (d) *FF* and (e) PCE of devices with different ETLs (The devices were prepared by two-step method Two-step solution process).

中国测试技术研究院
National Institute of Measurement and Testing Technology

检测报告
Test Report

中国认可 国际互认 检测 TESTING CNAS L0893

报告编号：检测字第 202505100834 号
Report No.

防伪码
d681a048f4b34405
b343fc6b75e96a5b
786b0511aa7e46e3
bb7d05ca14935e28

样品名称 Sample Name	Perovskite solar cells
标称生产单位 Manufacturer	Nanjing University of Aeronautics and Astronautics
委托单位 Client	Nanjing University of Aeronautics and Astronautics
联络信息 Contact Information	No. 29, Yudao Street, Qinhuai District, Nanjing, Jiangsu Province, China
检测类别 Test Category	Commission test

中国测试技术研究院 检测专用章
（检测专用章） Stamp

1010273555

授权签字人 Approved by 陈满满

签发日期 Issue Date 2025 年 Year 05 月 Month 29 日 Day

地址：中国·四川·成都玉双路 10 号
Address: No.10, Yushuang Road, Chengdu, Sichuan, China
邮编：610021
Post Code
网址：www.nimtt.cn
Web
电话：028-60828828
Telephone
传真：028-84404149
Fax
邮箱：kfzx@nimtt.com
E-mail

中国测试技术研究院检测报告 报 告 编 号 检测字第 202505100834 号
Test Report of NIMTT Report No.

检 测 结 果

Results of Test

1. Test Condition

Reference Cell: Mono-Si solar cell (window material: KG1).

Sample Information: Perovskite Solar Cells. Dimensions:(2.5×2.5) cm². Aperture area:0.0969 cm².

Storage Condition of Sample Before Test: Temperature: 25±5℃; humidity: 30±10%; stored in dark for 3 Days.

2. Methodologies and Settings

(1) In the 3A steady-state solar simulator (spectrum: AM1.5), the irradiance of the solar simulator was firs calibrated to 1000 W/m² with a standard solar cell, the temperature of the solar cell was controlled by a water bath thermostat at 25℃, and then the I-V parameters of the tested sample were measured with a digital source meter.

(2) The parameter Settings of the measurement software are shown in Table 1:

Table 1 Parameter Settings for I-V Test

Scan Mode	Start Voltage(V)	End Voltage(V)	Step (V)	Sweep point delay(ms)	Number of point	Light Soaking Pre-treatme nt
Reverse scan	1.3	-0.01	0.01	50	141	YES

3. Test Results

Current-voltage (I-V) curves are shown in Figures 1 and Table 2:

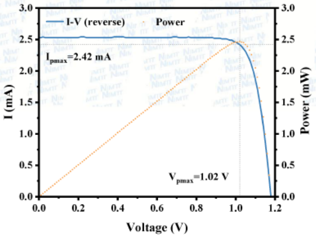

Figure 1 I-V Curve (Reverse Scan)

Table 2 I-V Parameter

Scan Mode	I_{sc} (mA)	V_{oc} (V)	FF (%)	P_{max} (mW)	V_{pmax} (V)	I_{pmax} (mA)	η (%)
Reverse scan	2.53	1.18	82.75	2.47	1.02	2.42	25.49

备注 Note: 1. Reported performance parameters take from one test value.
2. The solar cell area data is determined according to the mask area.

审核人员 Verified by 吴伟钢 主检人员 Tested by 康张李

续页专用（v202101） 第 4 页 共 4 页
Continued Page Page of

Supplementary Fig. 20. Device certification results in NIMTT (The device was encapsulated and equipped with an AR film).

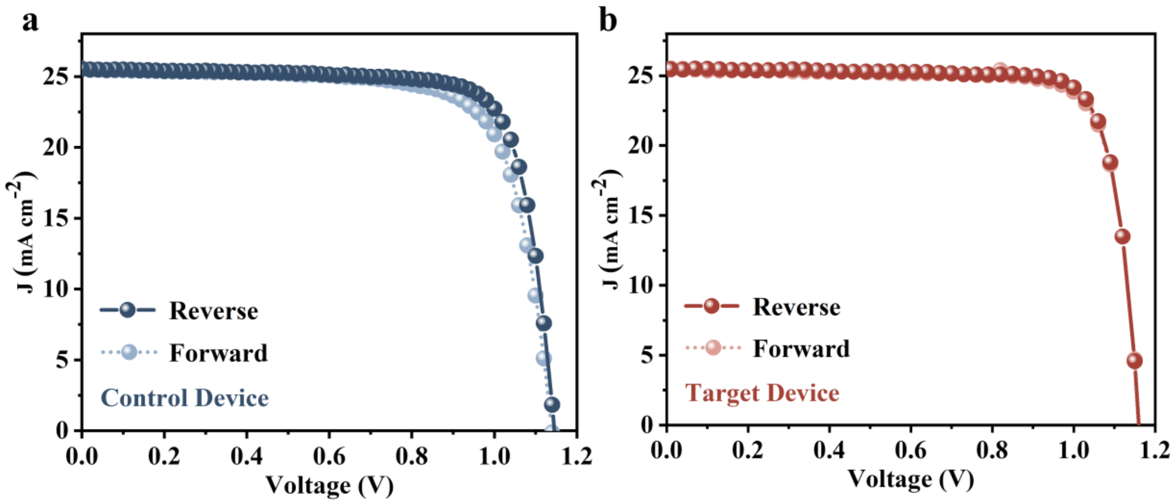

Supplementary Fig. 21. J-V characteristics of (a) control and (b) target devices along the reverse and forward scan directions.

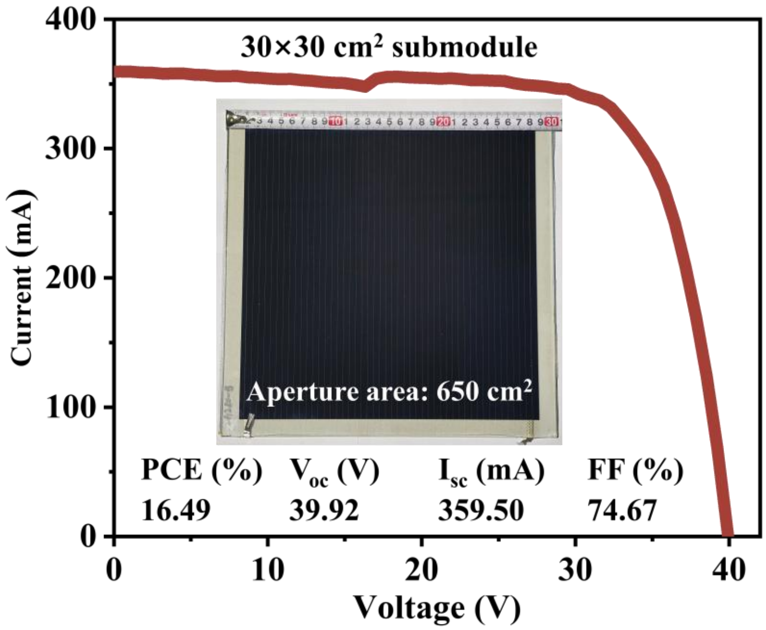

Supplementary Fig. 22. *J-V* curve and photograph (inset) of the 30 × 30 cm^2 target submodule.

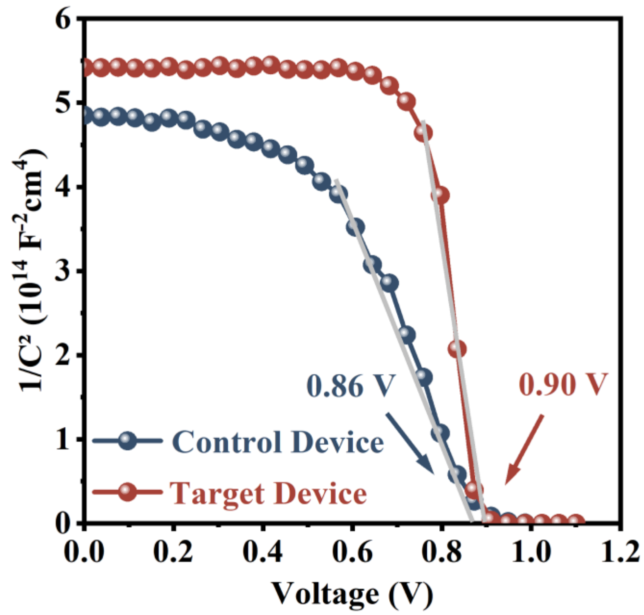

Supplementary Fig. 23. Mott-Schottky of control and target devices.

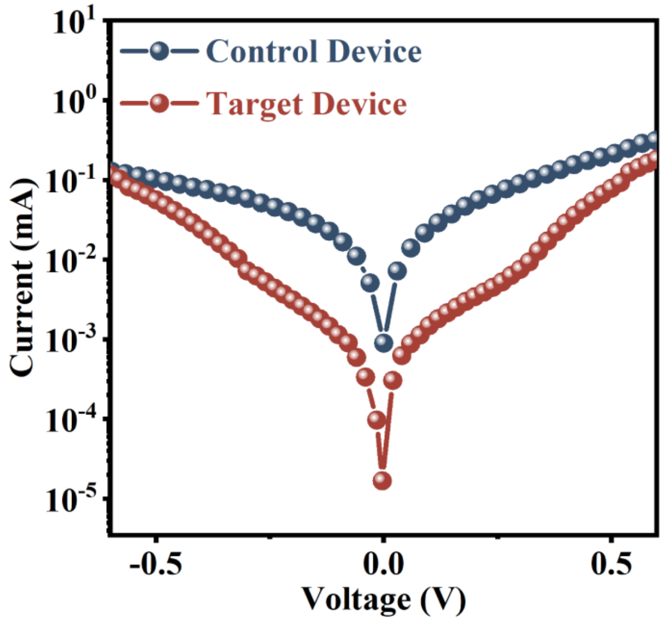

Supplementary Fig. 24. Dark *J*-*V* curves of control and target devices.

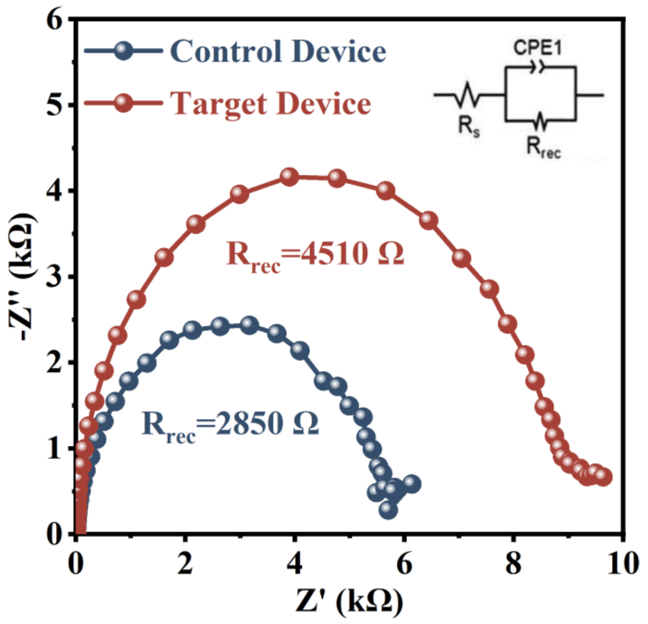

Supplementary Fig. 25. EIS of control and target devices.

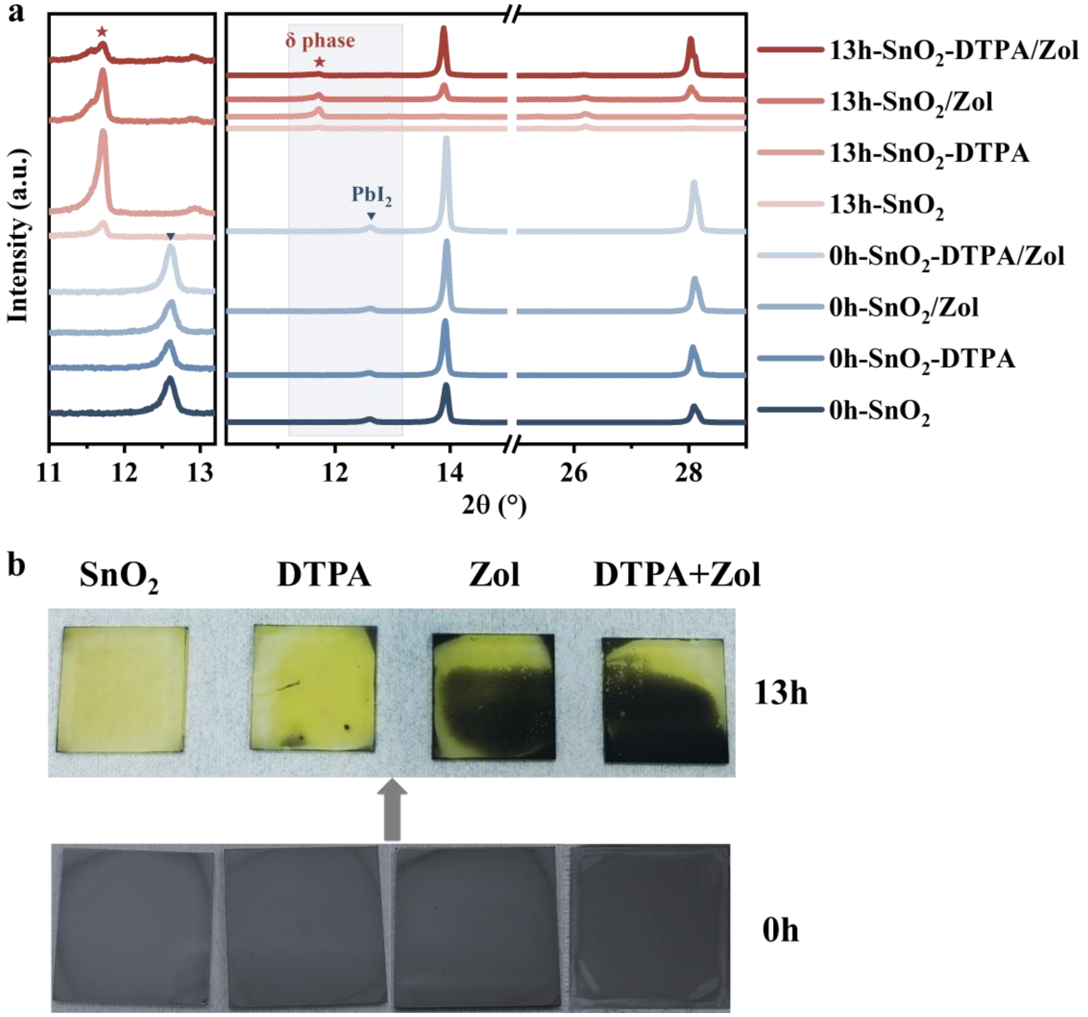

Supplementary Fig. 26. The results of UV light aging experiments for $FAPbI_3$ films grown on different SnO_2 substrates. (a) XRD patterns; (b) Photographs of the samples. (254nm UV, $T_{ambient}$=35°C, RH=85%).

After UV aging tests, the SnO_2-DTPA/Zol-based samples maintained a strong perovskite phase with only minimal δ-phase formation. The SnO_2/Zol-based samples exhibited more substantial δ-phase content while retaining partial perovskite phase stability. In contrast, the SnO_2-DTPA-based samples showed nearly complete perovskite phase degradation but preserved significant δ-phase components. The pure SnO_2-based samples demonstrated near-total signal loss with only trace δ-phase presence, suggesting almost complete decomposition into an amorphous state.

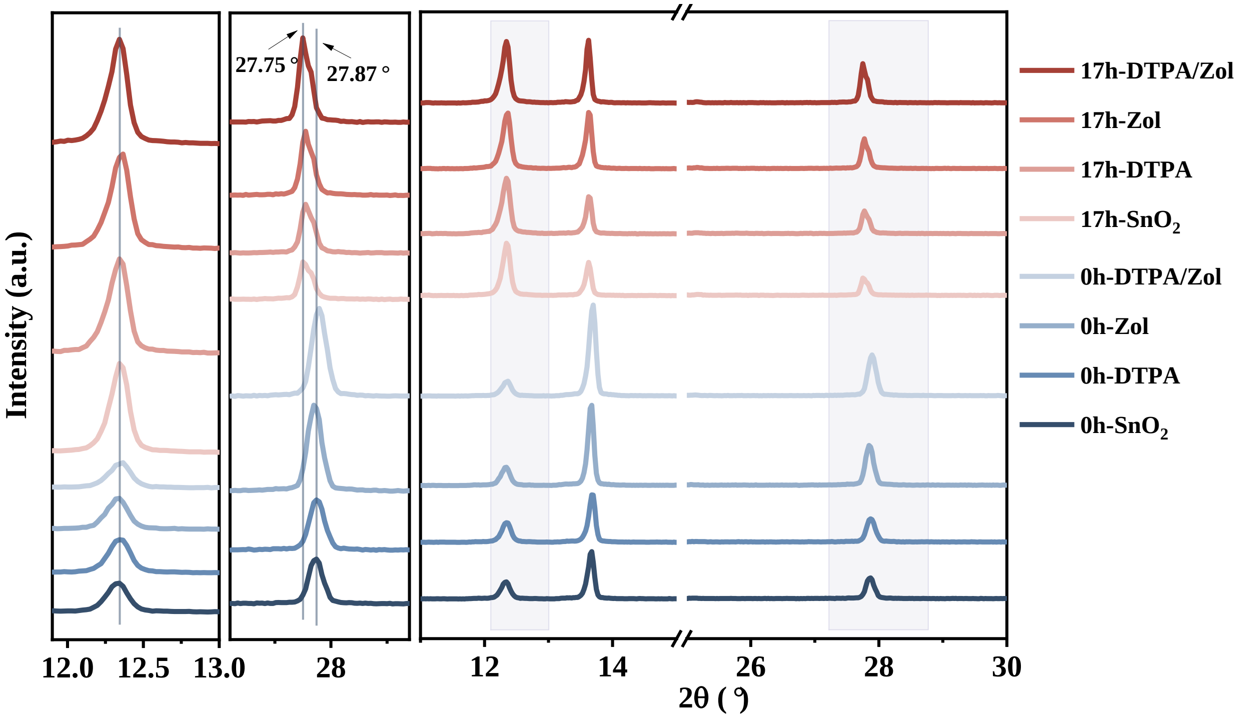

Supplementary Fig. 27. The XRD patterns of the damp heat test for perovskite films grown on different SnO_2 substrates. (110 °C Annealing, $T_{ambient}$=35 ℃, RH=85%, Natural Lighting).

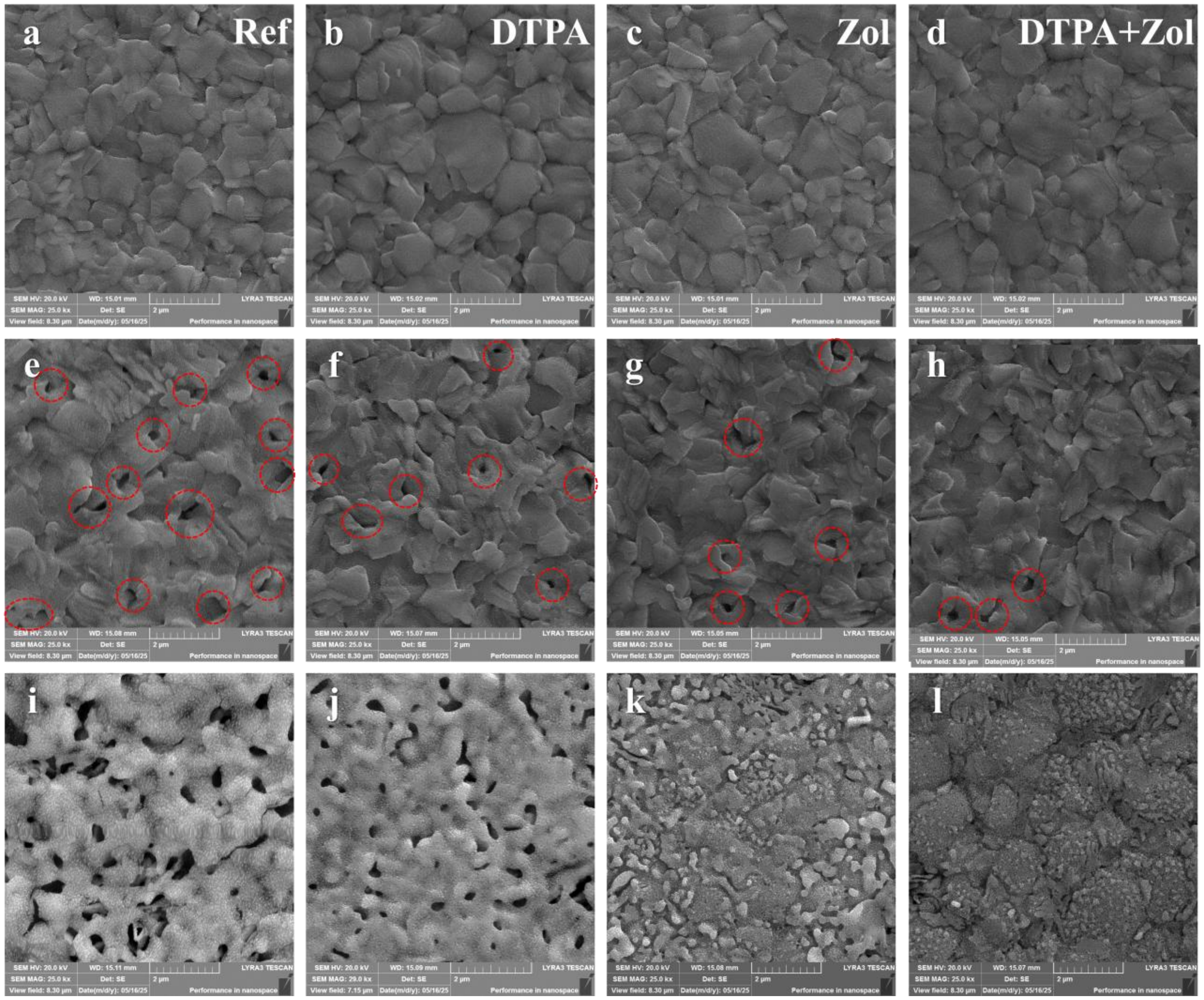

Supplementary Fig. 28. The SEM characterization of the films before and after aging tests.(a-d) SEM images of four samples before aging. (e-h) SEM images of four samples after thermal aging at 110 ℃ for 17 hours. (i-l) SEM images of four samples after UV light aging for 17 hours.

We conducted SEM characterization of the films before and after aging tests (Supplementary Fig. 28). Supplementary Fig. 28 a-d show SEM images of four samples before aging. Supplementary Fig. 28 e-h present SEM images of samples after thermal aging at 110 ℃ for 17 hours. The pure SnO_2 substrate samples exhibited numerous large pinholes, while the SnO_2-DTPA/Zol substrate samples showed significantly fewer pinholes. Supplementary Fig. 28 i-l display SEM images of samples after UV light aging for 17 hours. The perovskite films on pure SnO_2 substrates showed completely collapsed grain structures with extensive

pinholes and cracks, whereas films on SnO_2-DTPA/Zol substrates maintained relatively intact perovskite grain structures with only slight surface decomposition and no large pinholes or cracks.

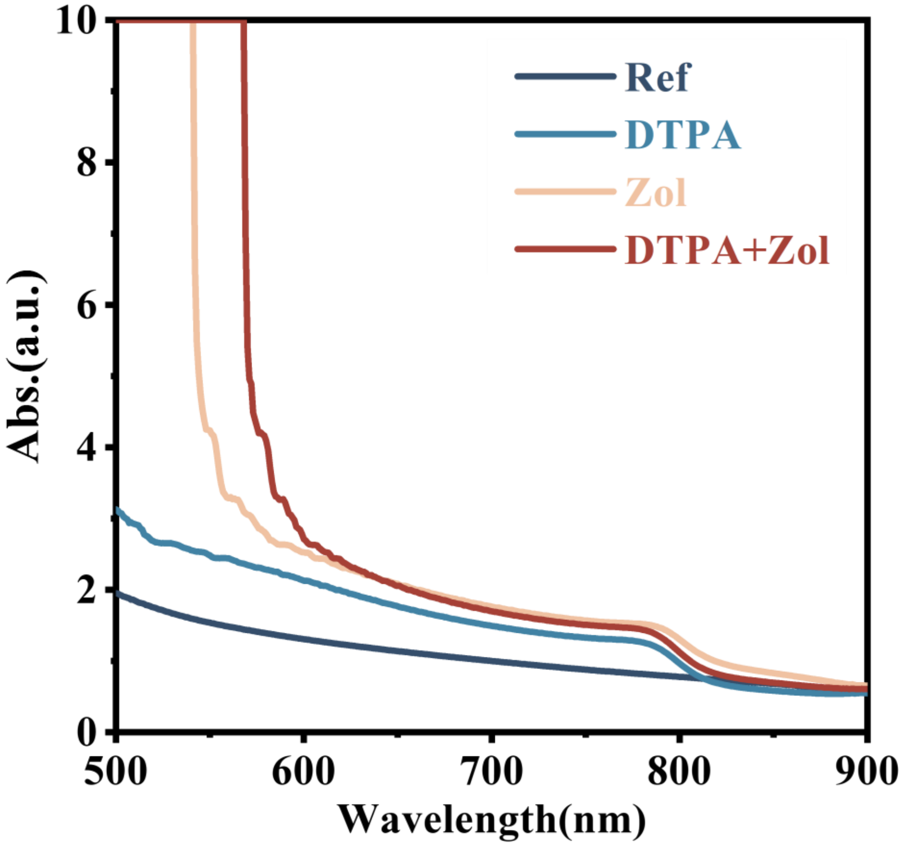

Supplementary Fig. 29. The UV-vis spectroscopy measurements on samples after 17 hours of UV exposure.

We performed absorption spectroscopy measurements on samples after 17 hours of UV exposure. As shown in Supplementary Fig. 29, the pure SnO_2 substrate samples demonstrated substantially reduced light absorption capability between 500-900 nm after aging, while the SnO_2-DTPA/Zol substrate samples maintained relatively strong light absorption capacity even after 17 hours of aging.

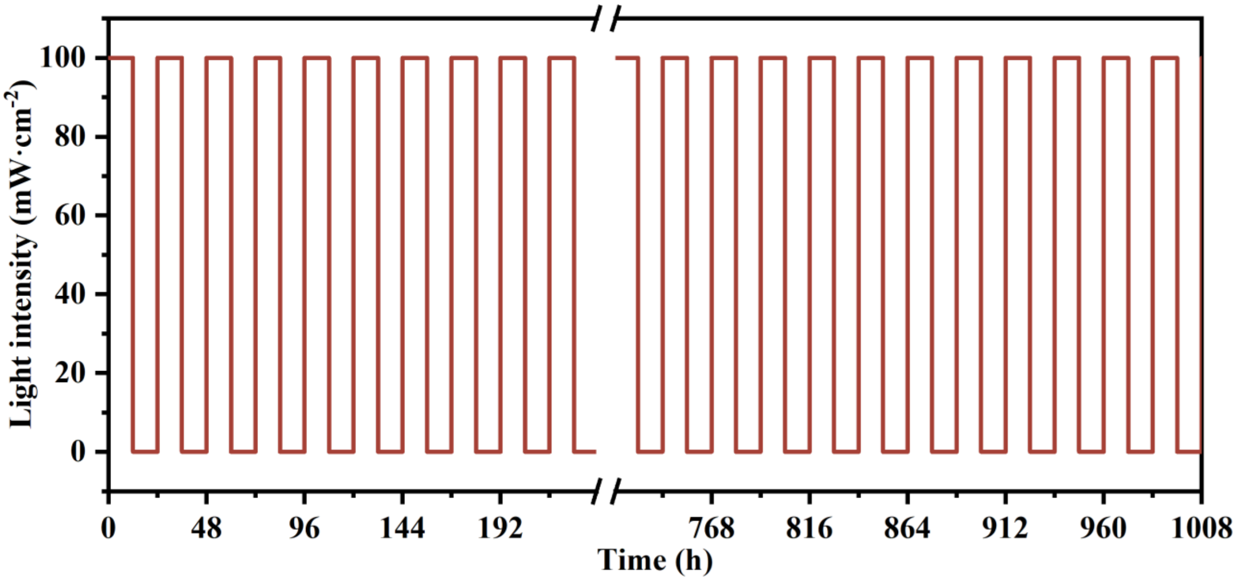

Supplementary Fig. 30. Light-dark cycling procedure of unencapsulated devices.

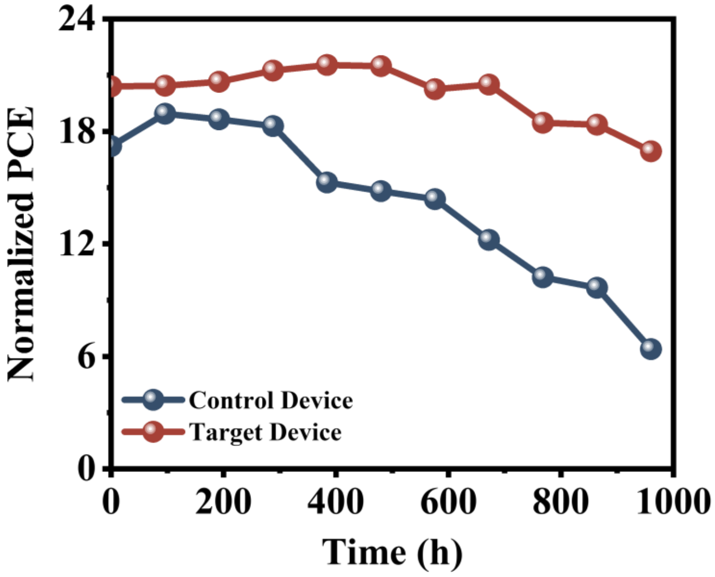

Supplementary Fig. 31. Efficiency evolution of PSCs under thermal cycling tests (24 h on/24 h off).

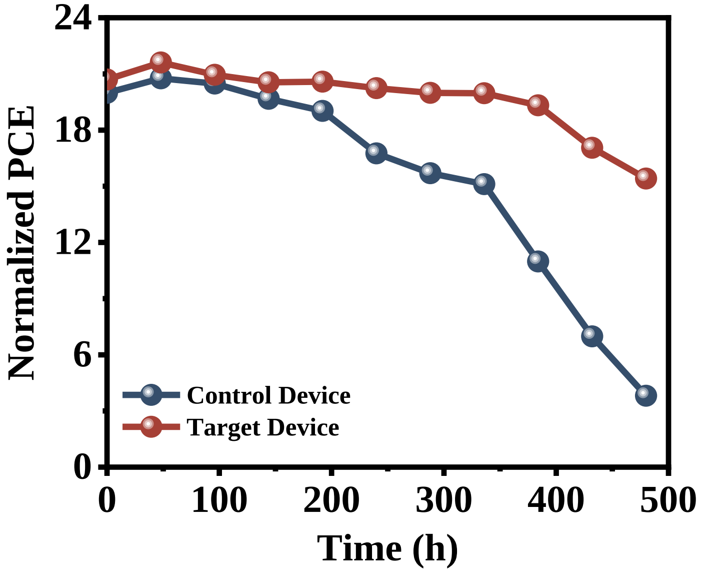

Supplementary Fig. 32. Efficiency evolution of PSCs under real outdoor storage tests.

Supplementary Table 1. The calculation data for relative content of KCl on SnO_2 substrate surface.

**Sample**	**DMF rinse**	**K 2p peak area**	**O 1s peak atomic ratio after peak fitting (%)**			**Ratio**
			**Lattice oxygen**	**Oxygen vacancies**	**-OH**	
SnO_2/KCl	Before	12195.71	75.08	19.04	5.88	18.98
SnO_2/KCl	After	4958.62	73.13	20.64	6.22	7.70

Supplementary Table 2. The calculation data for relative content of $CH_5N_3 \cdot HCl$ on SnO_2 substrate surface.

**Sample**	**DMF rinse**	**N 1s peak area**	**O 1s peak atomic ratio after peak fitting (%)**			**Ratio**
			**Lattice oxygen**	**Oxygen vacancies**	**-OH**	
SnO_2/Gua	Before	12807.32	71.71	21.18	3.75	22.41
SnO_2/Gua	After	8540.78	70.34	22.06	7.61	15.9

Supplementary Table 3. The calculation data for relative content of MAAc on SnO_2 substrate surface.

**Sample**	**DMF rinse**	**C 1s peak area**	**O 1s peak atomic ratio after peak fitting (%)**			**Ratio**
			**Lattice oxygen**	**Oxygen vacancies**	**-OH**	
SnO_2/MAAc	Before	7388.39	73.49	22.97	3.54	18.07
SnO_2/MAAc	After	6787.92	71.81	23.30	4.89	12.36

Supplementary Table 4. The calculation data for relative content of C-C, C-O/C-N and C=O on SnO_2 substrate surface.

Sample	Peak Name	Peak BE (eV)	Atomic conc (%)
SnO_2	C-C	284.80	69.43
	C-O/C-N	286.22	20.59
	C=O	288.68	9.98
SnO_2-DTPA	C-C	284.80	68.69
	C-O/C-N	286.28	22.28
	C=O	288.79	9.03
SnO_2/Zol	C-C	284.80	44.19
	C-O/C-N	286.35	49.05
	C=O	288.98	6.75
SnO_2-DTPA/Zol	C-C	284.80	44.71
	C-O/C-N	286.38	51.29
	C=O	288.95	4.01

Supplementary Table 5. The calculation data for the energy level of SnO_2, SnO_2-DTPA, SnO_2/Zol and SnO_2-DTPA/Zol films.

Sample	E_{cutoff} (eV)	E_{onset} (eV)	E_f (eV)	E_g (eV)	E_{VBM} (eV)	E_{CBM} (eV)
SnO_2	17.52	4.00	3.70	3.70	7.70	4.00
SnO_2-DTPA	17.51	3.91	3.71	3.70	7.62	3.92
SnO_2/Zol	17.26	3.92	3.96	3.70	7.88	4.18
SnO_2-DTPA/Zol	17.45	3.84	3.77	3.70	7.61	3.91

(E_{cutoff} , Secondary electron cutoff; E_{onset}, Secondary Electron Onset; E_f, Fermi energy level; E_g, Energy gap; E_{CBM}, conduction band minimum; E_{VBM}, valence band maximum.)

Supplementary Table 6. The calculation data for relative content of hydroxide on SnO_2 surface.

Sample	DMF rinse	Hydroxide peak area	Lattice oxygen peak area	Ratio
SnO_2	before	9990.22	94949.27	0.105216
	after	4778.38	44898.9	0.106425
SnO_2-DTPA	before	15807.03	129284.06	0.122266
	after	4466.85	48766.9	0.091596
SnO_2/Zol	before	11475.38	99222.8	0.115653
	after	3631.38	37871.36	0.095887
SnO_2-DTPA/Zol	before	12782.73	89557.91	0.142731
	after	7847.25	56760.21	0.138253

The formation of -OH groups on pure SnO_2 surfaces primarily occurs through two pathways: 1) Dissociation of adsorbed water molecules: When exposed to air, SnO_2 surfaces physically adsorb H_2O molecules, which may subsequently dissociate to form chemically adsorbed -OH and H^+ (H_2O + Sn-O-Sn → Sn-OH + Sn-OH). 2) Reaction of surface oxygen vacancies with water/oxygen: Oxygen vacancies (Vo) generated during SnO_2 film preparation can react with environmental H_2O or O_2 to form hydroxyl groups: a) Vo + H_2O → $2OH^-$; b) Vo + $1/2O_2$ + H_2O → $2OH^-$. Generally, more oxygen vacancies lead to higher surface hydroxylation[23-28].

In Figure 2f, the -OH peak area of pure SnO_2 remains nearly unchanged after DMF rinsing, indicating its surface chemical state is unaffected by DMF treatment. In Figures 2g and h, SnO_2-DTPA and SnO_2/Zol samples inherently contain more -OH groups than pure SnO_2 sample. This is because both DTPA and Zol molecules contain abundant -COOH and $-PO(OH)_2$ groups. During sample preparation, some of these -OH groups react and bond with the SnO_2 surface, while the remaining unbonded -OH groups increase the degree of surface hydroxylation. On the other hand, the modification of SnO_2 by -COOH and $-PO(OH)_2$ reduces the intrinsic defect concentration on the SnO_2 surface. Consequently, after extensive DMF washing, some

DTPA and Zol molecules are removed, leading to a decrease in -OH groups. The exposed defects, now fewer in number, undergo minimal hydroxylation. As a result, the -OH peak area of the washed SnO_2-DTPA and SnO_2/Zol samples becomes smaller than that of the pure SnO_2 sample.

Supplementary Table 7. Photovoltaic parameters of PSCs obtained along the reverse scan direction.

**PVK preparation method**	**Substrates**	V_{OC} **(V)**	J_{SC} **(mA/cm^2)**	***FF*** **(%)**	**PCE (%)**
Two-step solution process	SnO_2	1.118	25.45	75.61	21.51
	SnO_2-DTPA	1.138	25.57	77.20	22.46
	SnO_2/Zol	1.141	25.37	79.61	23.10
	SnO_2-DTPA/Zol	1.160	25.89	80.33	24.13
One-step solution process	SnO_2	1.14	24.12	82.50	22.65
	SnO_2-DTPA	1.15	24.80	82.96	23.66
	SnO_2/Zol	1.17	25.27	84.09	24.82
	SnO_2-DTPA/Zol	1.18	25.59	84.51	25.52

Supplementary Table 8. Summary of PCEs of high-efficiency (PCE > 24.5%) devices prepared in a full-air environment.

Perovskite	Relative Humidity (%)	Champion PCE (%)	Device structure	References
**$FAPbI_3$**	**20-50**	**25.52 (certificated 25.49)**	**n-i-p**	**This Work**
$FAPbI_3$	20	24.7	n-i-p	Y. Zou, *Science*, 2024[1]
$FAPbI_3$	30-85	25.74 (certificated 25.43)	n-i-p	Y.Yang, *Adv. Energy Mater.* 2024[2]
$Cs_{0.01}(FA_{0.97}MA_{0.03})_{0.99}Pb(I_{0.97}Br_{0.03})_3$	35-50	24.72	p-i-n	H. Meng, *Nature Energy*, 2024[3]

Supplementary Table 9. Fitted TRPL parameters for perovskite films with different ETLs.

**Sample**	A_1	A_2	τ_1 **(ns)**	τ_2 **(ns)**	τ_{ave} **(ns)**
FTO/SnO_2/Perovskite	1086.320	589.780	54.25	354.69	159.97
FTO/SnO_2-DTPA/Perovskite	248.050	257.890	41.46	246.01	145.72
FTO/SnO_2/Zol/Perovskite	519.560	310.800	32.11	252.43	114.57
FTO/SnO_2-DTPA/Zol/Perovskite	391.540	237.590	24.48	199.22	90.47

The PL decay fitting curve is based on the bi-exponential decay equation: $f(t)=A_1 \exp\left(\frac{-t}{\tau_1}\right)+A_2 \exp\left(\frac{-t}{\tau_2}\right)+y_0$.

Where A_1 and A_2 represent the decay amplitude, τ_1 represents trap-assisted recombination, τ_2 represents free carrier recombination, and y_0 is a constant for baseline offset.

Supplementary Table 10. Summary of dark storage stability of high-efficiency (PCE > 22%) devices.

Lifetime (hours)	Temperature (˚C)	Relative Humidity (%)	References
**T_{98} = 2750**	**24**	**35**	**This Work**
**T_{80} = 27000**	**24**	**35**	**This Work**
T_{98} = 1500	25	in N_2	D. Koo, *Nat. Nanotechnol.* 2024[4]
T_{95} = 1938	25	30-40	C. Ma, *Science*, 2023[5]
$T_{95.8}$ = 1000	25	25	J. W. Song, *Adv. Energy Mater.*, 2024[6]
$T_{95.1}$ = 2000	25	30-40	Y. Wang, *Adv. Mater.*, 2024[7]
$T_{91.8}$ = 4000	25	Ambient	X. Zhuang, *Adv. Mater.*, 2024[8]
$T_{95.1}$ = 1440	25	in N_2	Y. Zhang, *Angew. Chem. Int. Ed*, 2024[9]
$T_{88.3}$ = 350	25	45	F. Wang, *Adv. Mater.*, 2024[10]
$T_{94.7}$ = 1500	25	40	F. Wang, *Adv. Mater.*, 2024[10]
$T_{90.6}$ = 3552	25	in N_2	J. Cao, *Energy Environ. Sci.*, 2024[11]
T_{96} = 3000	25	45	Y. Yang, *Nature Energy*, 2024[12]

Supplementary Table 11. Summary of thermal stability of high-efficiency (PCE > 22%) devices.

Lifetime (hours)	Temperature (˚C)	Relative Humidity (%)	References
**T_{98} = 940**	**85**	**35**	**This Work**
**T_{80} = 19000**	**85**	**35**	**This Work**
T_{95} = 1600	85	in N_2	C. Liu, *Science*, 2023[13]
T_{95} = 1500	85	Ambient	H. Chen, *Science*, 2024[14]
T_{90} = 1500	85	Ambient (oven)	C. Li, *Science*, 2023[15]
$T_{91.1}$ = 1225	85	＜10% RH (in dry air)	Z. Huang, *Nature*, 2023[16]
T_{96} = 600	85	in N_2	C. Luo, *Nat. Photon.*, 2023[17]
$T_{83.5}$ = 1000	65	25% RH	J. W. Song, *Adv. Energy Mater.*, 2024[6]
T_{96} = 500	65	20-30% RH	Q. Zhao, *J. Am. Chem. Soc.*, 2024[18]
$T_{90.94}$ = 500	85	10-20% RH	X. Zhuang, *Adv. Mater.*, 2024[8]
T_{93} = 200	85	in N_2	Y. Zhang, *Angew. Chem. Int. Ed*, 2024[9]
T_{94} = 1000	85	in N_2	Y. Yang, *Nature Energy*, 2024[12]
T_{80} = 300	80	in N_2	H. Aqoma, *Nat Energy*, 2024[19]

Supplementary Table 12. Summary of light-dark cycling stability of high-efficiency (PCE > 22%) devices.

Lifetime (hours)	Temperature (˚C)	Relative Humidity (%)	References
**$T_{91.3}$ = 1008**	**~55℃-RT**	**35**	**This Work**
**T_{80} = 2600**	**~55℃-RT**	**35**	**This Work**
T_{80} = 1032	~55℃-RT	Ambient	Y. Shen, *Nature*, 2024[20]
T_{80} = 550	30	30	Y. Wang, *Adv. Mater.*, 2024[21]
T_{87} = 768	Ambient	Ambient	C. Tian, *Adv. Mater.*, 2024[22]

Supplementary References

1. Zou, Y. *et al.* A crystal capping layer for formation of black-phase $FAPbI_3$ perovskite in humid air. *Science* **385**, 161-167 (2024).
2. Yang, Y. *et al.* Compatible Soft-Templated Deposition and Surface Molecular Bridge Construction of SnO_2 Enable Air-Fabricated Perovskite Solar Cells with Efficiency Exceeding 25.7%. *Adv. Energy Mater* **14**, 2400416 (2024).
3. Meng, H. *et al.* Inhibition of halide oxidation and deprotonation of organic cations with dimethylammonium formate for air-processed p-i-n perovskite solar cells. *Natural Energy* **9**, 536-547 (2024).
4. Koo, D. *et al.* Mesoporous structured MoS_2 as an electron transport layer for efficient and stable perovskite solar cells. *Nat. Nanotechnol.* (2024) https://doi:10.1038/s41565-024-01799-8.
5. Ma, C. *et al.* Unveiling facet-dependent degradation and facet engineering for stable perovskite solar cells. *Science* **379**, 173-178 (2023).
6. Song, J. W. *et al.* Post-treated polycrystalline SnO_2 in perovskite solar cells for high efficiency and quasi-steady-state-IV stability. *Adv. Energy Mater.* **14**, 2401753 (2024).
7. Wang, Y. *et al.* Highly crystalized Cl-doped SnO_2 nanocrystals for stable aqueous dispersion toward high-performance perovskite photovoltaics. *Adv. Mater.* **36**, 2305849 (2024).
8. Zhuang, X. *et al.* Bottom-up defect modification through oily-allicin modified buried interface achieving highly efficient and stable perovskite solar cells. *Adv. Mater.* **36**, 2403257 (2024).
9. Zhang, Y. *et al.* An MBene modulating the buried SnO_2/perovskite interface in perovskite solar cells. *Angew Chem Int Ed* **63**, e202404385 (2024).
10. Wang, F. *et al.* Two-step perovskite solar cells with >25% efficiency: Unveiling the hidden bottom surface of perovskite layer. *Adv. Mater.* **36**, 2401476 (2024).

11. Cao, J. *et al.* Chlorinated-$Ti_3C_2T_F$ as a dual-functional buried interface on SnO_2 electron-transporting layers for 25.09% high-performance n-i-p perovskite solar cells. *Energy Environ. Sci.* **17**, 3454-3469 (2024).

12. Yang, Y. A thermotropic liquid crystal enables efficient and stable perovskite solar modules. *Nature Energy* **9**, 316-323 (2024).

13. Liu, C. *et al.* Bimolecularly passivated interface enables efficient and stable inverted perovskite solar cells. *Science* **382**, 810-815 (2023).

14. Chen, H. *et al.* Improved charge extraction in inverted perovskite solar cells with dual-site-binding ligands. *Science* **384**, 189-193 (2024).

15. Li, C. *et al.* Rational design of Lewis base molecules for stable and efficient inverted perovskite solar cells. *Science* **379**, 690-694 (2023).

16. Huang, Z. *et al.* Anion-π interactions suppress phase impurities in $FAPbI_3$ solar cells. *Nature* **623**, 531-537 (2023).

17. Luo, C. *et al.* Engineering the buried interface in perovskite solar cells via lattice-matched electron transport layer. *Nat. Photon.* **17**, 856-864 (2023).

18. Zhao, Q. *et al.* Oxygen vacancy mediation in SnO_2 electron transport layers enables efficient, stable, and scalable perovskite solar cells. *J. Am. Chem. Soc.* **146**, 19108-19117 (2024).

19. Aqoma, H. *et al.* Alkyl ammonium iodide-based ligand exchange strategy for high-efficiency organic-cation perovskite quantum dot solar cells. *Natural Energy* **9**, 324-332 (2024).

20. Shen, Y. *et al.* Strain regulation retards natural operation decay of perovskite solar cells. *Nature* **635**, 882-889 (2024).

21. Wang, Y. *et al.* Ordered perovskite structure with functional units for high performance and stable solar cells. *Adv. Mater.* **36**, 2401416 (2024).

22. Tian, C. *et al.* Air-processed efficient perovskite solar cells with full lifecycle management. *Adv. Mater.* 2411982 (2024). https://doi.org/10.1002/adma.202411982.

23. Batzill, M. *et al.* The surface and materials science of tin oxide. *Prog. Surf. Sci.* **79**, 47-154 (2005).

24. Henderson, MA. The interaction of water with solid surfaces: fundamental aspects revisited. *Surf. Sci. R.* **46**, 1-308 (2002).

25. Diebold, U. The surface science of titanium dioxide. *Surf. Sci. R.* **48**, 53-229 (2003).

26. Wu, ZY. *et al.* Periodic acid modification of chemical-bath deposited SnO_2 electron transport layers for perovskite solar cells and mini modules. *Adv. Sci.* **10**, 2300010 (2023).

27. Park, SY. *et al.* Advances in SnO_2 for efficient and stable n-i-p perovskite solar cells. *Adv. Mater.* **34**, 2110438 (2022).

28. Yun, HS. *et al.* Surface engineering of Tin Oxide nanoparticles by PH modulation facilitates homogeneous film formation for efficient perovskite solar modules. *Adv. Energy Mater.* **14**, 2400791 (2024).
