# Supplementary material for: Fully chemical interface engineering for statically and dynamically stable perovskite solar cells
Source: Nat Commun. 2025 Sep 29;16:8575. doi: 10.1038/s41467-025-63588-8 (PMC12480934; doi:10.1038/s41467-025-63588-8)
Supplement: Supplementary file 2 — Reporting Summary [file 41467_2025_63588_MOESM2_ESM.pdf]

Corresponding author(s): *Double-anonymous peer review submissions: write DAPR and your manuscript number here instead of author names.*

Solar Cells Reporting Summary

Nature Portfolio wishes to improve the reproducibility of the work that we publish. This form is intended for publication with all accepted papers reporting the characterization of photovoltaic devices and provides structure for consistency and transparency in reporting. Some list items might not apply to an individual manuscript, but all fields must be completed for clarity.

For further information on Nature Research policies, including our data availability policy, see Authors & Referees.

Experimental design

Please check the following details are reported in the manuscript, and provide a brief description or explanation where applicable.

1. Dimensions

Area of the tested solar cells

☒ Yes — Aperture areas of 0.04, 0.094 and 0.28 cm2 were used in this work.
☐ No — *Explain why this information is not reported/not relevant.*

Method used to determine the device area

☒ Yes — Black metal aperture masks were used during the J-V measurements.
☐ No — *Explain why this information is not reported/not relevant.*

2. Current-voltage characterization

Current density-voltage (J-V) plots in both forward and backward direction

☒ Yes — Provided in this manuscript
☐ No

Voltage scan conditions

☒ Yes — Provided in this manuscript
☐ No — *Explain why this information is not reported/not relevant.*

Test environment

☒ Yes — Provided in this manuscript
☐ No — *Explain why this information is not reported/not relevant.*

Protocol for preconditioning of the device before its characterization

☐ Yes — *Provide a description of the protocol.*
☒ No — *Explain why this information is not reported/not relevant.*

Stability of the J-V characteristic

☒ Yes — Stabilized PCEs of single-junction solar cells were provided.
☐ No — *Explain why this information is not reported/not relevant.*

3. Hysteresis or any other unusual behaviour

Description of the unusual behaviour observed during the characterization

☒ Yes — Very minor hysteresis was observed for devices herein.
☐ No — *Explain why this information is not reported/not relevant.*

Related experimental data

☒ Yes — J-V curves under reverse and forward scans were provided.
☐ No — *Explain why this information is not reported/not relevant.*

4. Efficiency

External quantum efficiency (EQE) or incident photons to current efficiency (IPCE)

☒ Yes — EQE curves were provided.
☐ No — *Explain why this information is not reported/not relevant.*

A comparison between the integrated response under the standard reference spectrum and the response measure under the simulator

☒ Yes — The integrated Jsc values from EQE were consistent with Jsc values from J-V measurements.
☐ No — *Explain why this information is not reported/not relevant.*

For tandem solar cells, the bias illumination and bias voltage used for each subcell

☐ Yes
☒ No

Provide a description of the measurement conditions.

Explain why this information is not reported/not relevant.

5. Calibration

Light source and reference cell or sensor used for the characterization

☒ Yes
☐ No

Stated in Method section.

Explain why this information is not reported/not relevant.

Confirmation that the reference cell was calibrated and certified

☐ Yes
☒ No

Identify the independent certification laboratory.

Explain why this information is not reported/not relevant.

Calculation of spectral mismatch between the reference cell and the devices under test

☐ Yes
☒ No

The light spectrum used for measurements matches well with the reference silicon cell, and we did not calculate the spectral mismatch between the reference cell and the tested devices

Explain why this information is not reported/not relevant.

6. Mask/aperture

Size of the mask/aperture used during testing

☒ Yes
☐ No

Metal aperture masks with areas of 0.04 and 0.094 cm2 were used for testing.

Explain why this information is not reported/not relevant.

Variation of the measured short-circuit current density with the mask/aperture area

☐ Yes
☒ No

We measured all devices with masks.

Explain why this information is not reported/not relevant.

7. Performance certification

Identity of the independent certification laboratory that confirmed the photovoltaic performance

☐ Yes
☒ No

Identify the independent certification laboratory.

Explain why this information is not reported/not relevant.

A copy of any certificate(s)

☐ Yes
☒ No

Certificate copies should be provided in the Supplementary information. Please state the supplementary item number.

Explain why this information is not reported/not relevant.

8. Statistics

Number of solar cells tested

☒ Yes
☐ No

Stated in the manuscript.

Explain why this information is not reported/not relevant.

Statistical analysis of the device performance

☒ Yes
☐ No

Stated in the manuscript.

Explain why this information is not reported/not relevant.

9. Long-term stability analysis

Type of analysis, bias conditions and environmental conditions

☒ Yes
☐ No

Stated in the manuscript.

Explain why this information is not reported/not relevant.
